# Health Professionals’ Perspectives on Electronic Medical Record Infusion and Individual Performance: Model Development and Questionnaire Survey Study

**DOI:** 10.2196/32180

**Published:** 2021-11-30

**Authors:** Rai-Fu Chen, Ju-Ling Hsiao

**Affiliations:** 1 Department of Information Management Chia-Nan University of Pharmacy and Science Tainan City Taiwan; 2 Department of Pharmacy Chia-Nan University of Pharmacy and Science Tainan City Taiwan

**Keywords:** health care professional, electronic medical records, IS infusion, individual performance, EHR, electronic health record, performance, perspective, information system, integration, decision-making, health information exchange, questionnaire

## Abstract

**Background:**

Electronic medical records (EMRs) are integrated information sources generated by health care professionals (HCPs) from various health care information systems. EMRs play crucial roles in improving the quality of care and medical decision-making and in facilitating cross-hospital health information exchange. Although many hospitals have invested considerable resources and efforts to develop EMRs for several years, the factors affecting the long-term success of EMRs, particularly in the EMR infusion stage, remain unclear.

**Objective:**

The aim of this study was to investigate the effects of technology, user, and task characteristics on EMR infusion to determine the factors that largely affect EMR infusion. In addition, we examined the effect of EMR infusion on individual HCP performance.

**Methods:**

A questionnaire survey was used to collect data from HCPs with >6 months experience in using EMRs in a Taiwanese teaching hospital. A total of 316 questionnaires were distributed and 211 complete copies were returned, yielding a valid response rate of 66.8%. The collected data were further analyzed using WarpPLS 5.0.

**Results:**

EMR infusion (*R^2^*=0.771) was mainly affected by user habits (*β*=.411), portability (*β*=.217), personal innovativeness (*β*=.198), technostress (*β*=.169), and time criticality (*β*=.168), and individual performance (*R^2^*=0.541) was affected by EMR infusion (*β*=.735). This finding indicated that user (habit, personal innovativeness, and technostress), technology (portability), and task (mobility and time criticality) characteristics have major effects on EMR infusion. Furthermore, the results indicated that EMR infusion positively affects individual performance.

**Conclusions:**

The factors identified in this study can extend information systems infusion theory and provide useful insights for the further improvement of EMR development in hospitals and by the government, specifically in its infusion stage. In addition, the developed instrument can be used as an assessment tool to identify the key factors for EMR infusion, and to evaluate the extent of EMR infusion and the individual performance of hospitals that have implemented EMR systems. Moreover, the results can help governments to understand the urgent needs of hospitals in implementing EMR systems, provide sufficient resources and support to improve the incentives of EMR development, and develop adequate EMR policies for the meaningful use of electronic health records among hospitals and clinics.

## Introduction

### Background

Electronic medical records (EMRs), as an important health information technology (HIT), have been developed to solve problems arising from the use of paper medical records, including the difficulty in searching for information, incompleteness of information, illegibility of handwriting, difficulty in management and storage, and inaccessibility [1]. EMRs are computerized medical information systems that collect, store, and display patient information generated by health care professionals (HCPs) from various health care information systems, including hospital information systems (HISs), picture archiving and communication systems, laboratory information systems, radiology information systems, and others [2]. EMR systems can be regarded as both electronic health record (EHR) systems and clinical information systems to provide clinical (including patient history and clinical notes, prescription management and patient demographics, and patient care management), communicational (visualization of results, communication with other institutions, and electronic transfers), and administrative (appointments scheduling and distance access, and billing and data security) functionalities [3]. Therefore, EMRs play a crucial role in improving the care quality, continuity, safety, efficiency, and decision-making in health care, and facilitate the cross-hospital exchange of health information [1,2,4-9].

Prior studies found positive correlations between information technology (IT)/information system (IS) utilization and individual and organizational performance [10-12]. With the wide-ranging use of HITs in the health care industry, understanding the usage behavior of HCPs is an important research topic for further HIT development. Underutilization of a system is often a major problem contributing to lack of complete infusion or integration of the implemented IS into employees’ daily work or organizational processes after it is introduced [13,14]. Underutilization of a system has been considered as one of the key causes for a system not meeting the initial expectations in increasing productivity and yielding reasonable returns [15-17]. IS infusion, the final stage of the IS development process in Cooper and Zmud’s [18] IS implementation model, is defined as using the system to its full potential in an extended, integrative, and emergent way. Organizations can fully leverage their investments in IS infusion because users voluntarily go beyond standardized system usage and exploit the system’s full potential to improve their task performance [19,20]. Despite being recognized as critical to the long-term success of an IS, particularly for full realization of its potential [19], relatively little attention has focused on how infusion occurs [3,14,21-23]. In addition, some studies argued that most IS infusion studies have mainly focused on technological aspects at an organizational level rather than an individual level [22-24]. This is a problem as the results obtained from such studies cannot offer useful suggestions and improvements to major system users for further explorative, integrative, and future use, and may further cause negative effects in individual and organizational performance derived from use of the IT/IS.

Although EMRs can provide clinical and operational benefits, EMR adoption is lagging because of user resistance and other barriers [2]. Most EMR-related studies have mainly focused on issues that arise in the early stage of EMR development, particularly for investigating the factors or barriers affecting EMR adoption or acceptance [2,9,25-32]. Trudel et al [33] found that an increase in EMR adoption does not lead to physicians’ progress in using EMR systems during EMR assimilation, which are the routinization and infusion stages mentioned by Cooper and Zmud [18]. Raymond et al [34] called for a deeper understanding of the factors leading to greater performance outcomes from EMR systems after extended EMR use. Moreover, Bhattacherjee [35] confirmed that the long-term success of an IT/IS depends on its continued use rather than its first use, and the influencing factors toward the use of an IT/IS may vary in various IT/IS implementation stages. Ng and Kim [36] argued that IS infusion requires the authentic motivation of users, which is not the case for IS adoption and continuance, and they indicated a lack of understanding about the authentic motivations leading to infusion in the existing literature. Due to the paucity of HIT infusion studies [21,22], the factors (motivations) concerning EMR infusion in the context in which EMR systems are being integrated in clinical settings and incorporated in routine practice should be evaluated. Identification and understanding of the key determinants and consequents of EMR infusion will be helpful to minimize the gaps between IT practitioners and HCPs in EMR design and implementation, and enable further improvements to meet organizational expectations.

Numbeo [37] reported that the health care system of Taiwan ranks first among 93 countries. The successful implementation of HIS and EMRs with full integration of various ITs/ISs in health care institutes is considered the key to the early success of cross-hospital exchange of EMRs in Taiwan. For example, during the COVID-19 pandemic, authorized HCPs in Taiwanese clinics or hospitals can request patient medical records within 3 months and check patients’ travel history, occupation, contact history, and cluster history using the patient’s health insurance card information to help reduce the infection risk to HCPs and to enable electronic transfer, if necessary, for providing better patient care. Although many Taiwanese hospitals have invested substantial resources and efforts to develop EMRs, with long-term government support for eHealth and EMR development since 2009, factors that affect the long-term success of EMR infusion and performance remain unclear. This situation calls for further research from a practical perspective to provide significant insights for EMR development, particularly in the EMR infusion stage, and the results may further provide contributions to HIT development.

Moreover, the initial success of EMR adoption and acceptance does not ensure its long-term success in terms of its incorporation into the daily operating procedures of hospitals in the technology infusion stage. Therefore, the purpose of this study was to understand the determinants and consequences (performance impacts) of EMR infusion at the individual level. The research questions addressed in this study were: (1) What are the salient factors (motivations) of technology, task, and user influencing EMR infusion by HCPs? (2) How do these factors (motivations) influence EMR infusion and performance?

### Prior Related Studies

#### IT Infusion and Performance

ITs and ISs have the potential to substantially improve the operational effectiveness and efficiency of an organization if used appropriately. IT implementation is a dynamic IT adaption process in organizations with various stages, including initiation, adoption, adaptation, acceptance, routinization, and infusion, as identified by Cooper and Zmud [18] based on a technological diffusion approach. This general IT implementation model has been widely accepted and used in the IS discipline by various users and contexts for exploring the key factors influencing different implementation stages of specific systems. The IT postadoption stages, including IT acceptance, routinization, and infusion as defined by Cooper and Zmud [18], are often the main focus of such research because they are highly relevant to actual IT usage and organizations can observe the realized benefits obtained through IT usage [15,36].

Saga and Zmud [20] investigated the nature and determinants of IT acceptance, routinization, and infusion, and they identified key variables and determinants related to various IT postadoption stages. They found that standardized use, use perceived as “normal,” and administration infrastructure development are key characteristics of IT/IS routinization, whereas extended use, integrative use, and emergent use of IT/IS can be represented as the measurement variables for IT/IS infusion. For the IT implementation stages, IT assimilation and IT infusion are two relevant and similar concepts but with slight differences in nature and the applied theories. Armstrong and Sambamurthy [38] defined IT/IS assimilation as “the success achieved by firms in utilizing the capabilities of IT/IS to enhance their business performance.” IT/IS assimilation can bring significant business value if IT/IS becomes a routinized element of value-chain activities and business strategies in a firm [18,38]. They further argued that IT assimilation “focuses more on the extent of which IT has been infused into specific business activities” and “how effectively IT is enabling the conduct of those activities relative to rivals.” Thus, IT/IS assimilation can be viewed as a broader and integrative stage across IT/IS routinization and infusion stages [18].

Some well-known theoretical models have been proposed and validated for IT/IS postadoption stages, including technology adoption [17,39,40], IS continuance [35], and IS infusion [41,42]. Technology acceptance models (TAMs) are widely used for IT/IS evaluation in the technology adoption stage [17,39,40], whereas IS continuance models are used for evaluation in the IS routinization stage [35]. Ng and Kim [36] argued that most IS research has placed substantial attention on initial system adoption and continuance; however, only few IS infusion studies have been performed to date, which have produced mixed and inconclusive results due to the lack of consideration of the authentic motivation of users in IS infusion. Tennant et al [23] argued that existing implemented systems are often underutilized or not used effectively. They suggested focusing on a deeper level of usage (ie, the infusion stage) that can enhance work tasks and performance. Tennant et al [23] summarized the definitions of infusion used by researchers into two main types: (1) use of IT in a more comprehensive and integrated manner to support the higher-level aspects of organizational work, resulting in the use of IT at its full potential [18]; and (2) the extent to which the full potential of innovation has been embedded in an organization’s (or individual’s) work system [43].

Ng and Kim [36] investigated the relationships between user empowerment, which is regarded as the authentic motivation derived from psychological empowerment theory, and IS infusion. They also examined the moderating effect of habit between user empowerment dimensions and IS infusion types. The results showed that user empowerment dimensions have significant effects on the IS infusion types. In addition, the moderating role of habit between motivations of user empowerment and IS infusion (extended use and integrative use) was confirmed. O’Connor et al [22] and Tennant et al [23] argued that the majority of infusion studies have largely focused on technological aspects at an organizational level rather than an individual level. This may lead to difficulty in the extent to which the full potential of IT/IS can be embedded in an organization’s or individual’s work system. Tennant et al [23] proposed a conceptual research model based on concepts derived from the system usage perspective reported by Burton-Jones and Straub [44] and the task-technology fit (TTF) perspective described by Goodhue and Thompson [10] to examine IS infusion and performance. According to the research model, factors related to the characteristics of the system, individual or user, and task are the antecedents of IS infusion, which may subsequently affect an individual’s performance. Although the IS infusion model proposed by Tennant et al [23] provided a starting point to understand the nature of IS infusion and its effects on consequences, the model should be further validated in various contexts.

Performance evaluation is a key surrogate measure for IS success within the IS discipline in the postimplementation stage [10-12]. Goodhue and Thompson [10] proposed a TTF model by integrating the perspectives of fit and utilization focus to explore the antecedents (task, technology, and individual factors) of TTF, and the relationship between TTF, utilization, and individual performance. They reported that IT/IS can positively affect individual performance if the technology is used (utilization) and is a good fit for the supported task. Some studies have adopted the TTF model to investigate task, technology, and individual fit, and to explore their effects on individual performance [45,46]. By contrast, other studies have examined IT/IS models by incorporating the TTF construct across various contexts (user groups, technologies, and tasks) [47-49]. For example, Hsaio and Chen [45] examined the TTF of mobile nursing ISs for nursing performance on the basis of the TTF model. Dishaw and Strong [48] integrated two IT utilization models (TTF and TAM, as an extension of the TAM with the TTF construct) and found that the integrated model had more explanatory power than each individual model (TTF or TAM) and facilitated a more favorable understanding of IT utilization.

The TTF model has been considered as the core of investigating IS infusion and its effect on individual performance [21-23]. In a study based on the TTF perspective, Tennant et al [23] indicated that future studies related to IS infusion should incorporate the elements of user, task, and system, because IS infusion involves the use of technologies that assist individuals in performing their tasks. Hsaio and Chen [50] and O’Connor et al [21,22] proposed research models by incorporating user, task, and system characteristics as the determinants to investigate mobile health (mHealth) continuance and infusion based on the TTF perspective. In addition, Goodhue and Thompson [10] indicated that the feedback mechanism of performance may affect subsequent TTF, utilization, and performance. For the long-term evaluation of an IS, the fit among the task, technology, and individual should be evaluated, and the IS should be continuously used for supporting the tasks. Thus, this process of IS infusion may affect individual performance.

#### EMR-Related Studies

HIMSS Analytics defined an EMR as “an application environment composed of the clinical data repository, clinical decision support, controlled medical vocabulary, order entry, computerized provider order entry, pharmacy, and clinical documentation applications.” Moreover, the environment contains patients’ EMRs across inpatient and outpatient services, and is used by HCPs to document, monitor, and manage health care delivery within a health care organization [2]. Boonstra and Broekhuis [2] mentioned that EMRs are computerized medical information systems that collect, store, and display patient information. Yamamoto and Khan [9] indicated that the perceived advantages of EMRs include:

optimizing the documentation of patient encounters, improving the communication of information among physicians, improving access to patient medical information, reducing errors, optimizing billing, improving reimbursement for services, forming a data repository for research and quality improvement, and reducing the use of papers.

Furthermore, EMRs designed to document patient care information and enable data sharing among clinicians [29,51] can disrupt work practices, thus causing a significant decrease in productivity with their initial use [52,53].

EMR systems are viewed as both EHR systems and clinical information systems in hospital settings to provide clinical, communicational, and administrative functionalities [3]. Among the investigated three functionalities, Raymond et al [3] found that clinical functionalities can explain why certain primary care physicians make more extended use of EHRs than others because clinical functionalities can better support or fit their main medical tasks. The core components of an EHR are administrative functions, computerized physician order entry, lab systems, radiology systems, pharmacy systems, and clinical documentation [54]. Raymond et al [3] indicated that EHR systems have been slowly adopted by health care providers in the United States due to the challenges of costly software packages, system security, patient confidentiality, and unknown future government regulations.

Although the appropriate use of EMRs can improve quality, continuity, safety, and efficiency in health care, they have not been widely adopted in health care institutions as expected because of resistance among HCPs [2]. To understand the key barriers to the use of EMRs, Boonstra and Broekhuis [2] performed a comprehensive systematic review of studies related to the use of EMRs among physicians in the early stage of EMR development in health care institutions. They identified the following 8 key barriers affecting physicians’ acceptance of EMR implementation: organizational, process change, financial, technical, time, psychological, social, and legal barriers. O’Donnell et al [55] investigated primary care physicians’ attitudes toward EMR adoption and proposed a clinical adoption framework to understand the disparate performance among health care institutions in EMR implementation. Some studies have explored EMR adoption based on technological evaluation theories, namely the theory of planned behavior and the unified theory of acceptance and use of technology (UTAUT) [56-58]. Ahmed et al [56] investigated predicators of intention to use EMR based on the expanded UTAUT model (UTAUT2). They identified performance expectancy, effort expectancy, social influence, facilitating conditions, and computer literacy as key predicators of intention to use EMRs by health care providers; however, hedonic motivation and habit had no significant effect on intention to use EMRs. In addition, Sayyah Gilani et al [59] performed a study on EMR continuance intention of HCPs based on technology continuance theory. They found that EMR continuance was influenced by attitude and satisfaction. Attitude is mainly influenced by perceived ease of use, perceived usefulness, and satisfaction, whereas satisfaction is influenced by perceived usefulness and confirmation. In turn, perceived usefulness is affected by perceived ease of use and confirmation.

However, previous studies have argued that the scope of these theories is limited because they do not address the causes related to the adoption process, specifically for the postimplementation stage [28,57,60]. In addition, Trudel et al [33] reported the presence of the ceiling effect on EMR assimilation based on their observation that the growth of EMR adoption does not lead to physicians’ progress in using EMR systems. Raymond et al [34] called for a deeper understanding of the factors leading to greater performance outcomes from EMR systems after extended EMR use. Goh et al [61] proposed a dynamic process model based on adaptive structuration theory for improving understanding of the interplay between HIT and patterns of clinical work embodied in daily routines. O’Connor et al [21] investigated the determinants (factors adapted from technology, user, and task) of individual infusion of mHealth technologies and their subsequent outcomes. In their conceptual model, technology, user, and task were considered key enablers of successful mHealth infusion, and mHealth infusion led to improvements in preventative care, greater decision-making, and reduced medical errors. O’Connor et al [22] further proposed an mHealth infusion model to investigate the effects of the characteristics of technology (availability, maturity, and portability), individuals (habits, self-efficacy, and technology trust), and tasks (time criticality, interdependence, and mobility) on the integrative and exploratory use of mHealth infusion by HCPs and the relationship between mHealth infusion and performance.

Bhattacherjee [35] indicated that the long-term success of an IS depends on its continued use in the postimplementation stage, and proposed the expectation confirmation model (ECM) to understand IS continuance by examining the relationships among confirmation, perceived usefulness, satisfaction, and IS continuance. In addition, the author indicated that the key factors determined for IS acceptance are not necessarily crucial in the IS postimplementation or infusion stage. This finding implied that factors affecting ISs may exert different effects on different IS implementation stages. Hsaio and Chen [50] investigated the determinants of HCPs’ perspectives on mHealth continuance and performance based on the ECM proposed by Bhattacherjee [35] and the mHealth infusion model proposed by O’Connor et al [22]. They found that mHealth continuance is mainly affected by perceived usefulness, technology maturity, individual habits, task mobility, and user satisfaction, whereas individual performance is substantially affected by mHealth continuance.

The aforementioned findings provide the theoretical basis for this study in terms of understanding the effects of the antecedents of EMR infusion (task, technology, and individual characteristic) on the exploratory, integrative, and future (emergent) use of EMRs, and the subsequent effects on individual performance.

## Methods

### Research Model

**Figure 1:**
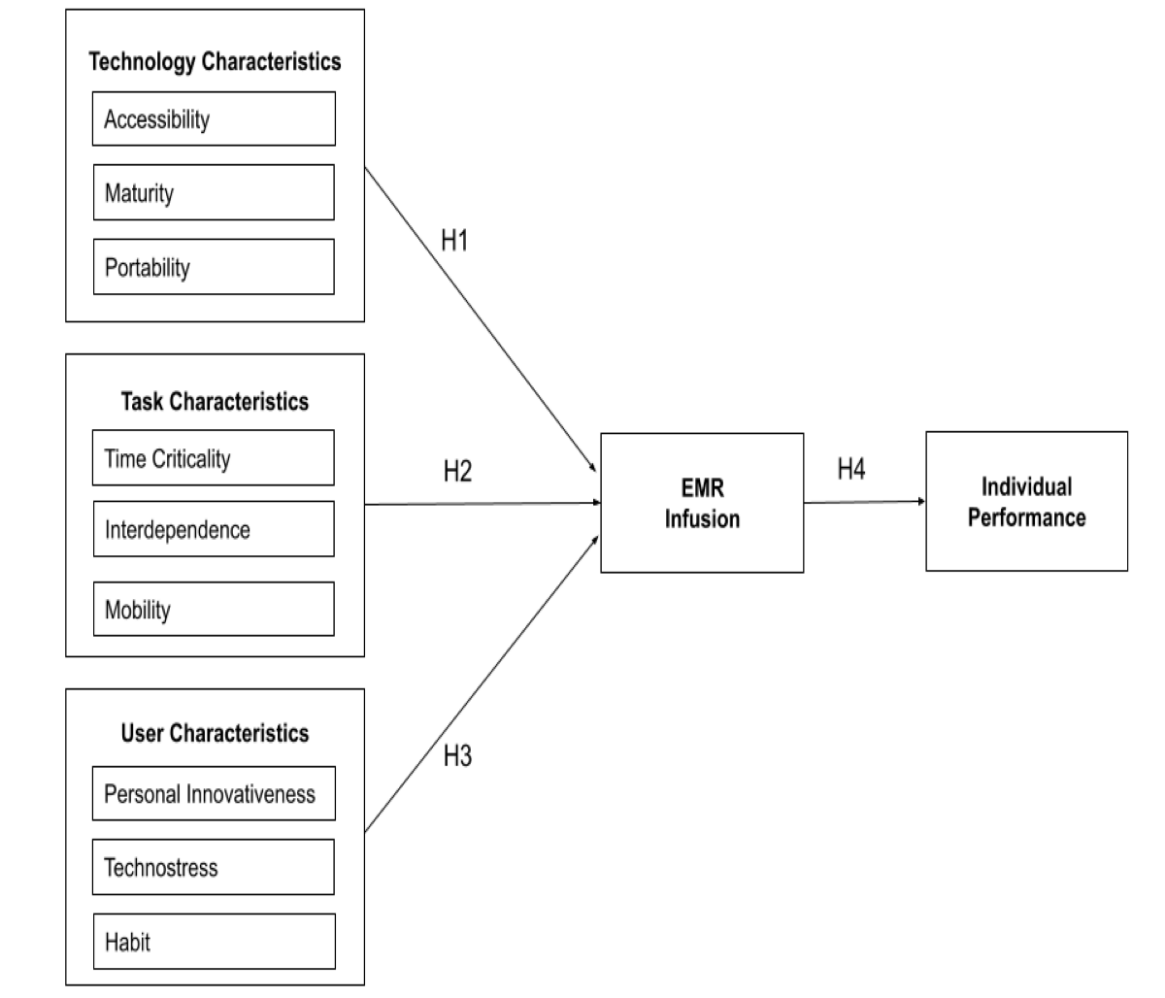
Research model. EMR: electronic medical record. H: hypothesis.

On the basis of the results reported by Hsaio and Chen [50] and O’Connor et al [22] regarding mHealth infusion, and those by Goodhue and Thompson [10] regarding the TTF model, we constructed an EMR infusion model to investigate the effects of technology, user, and task characteristics on EMR infusion by HCPs and their subsequent effects on individual performance. In addition, some variables mentioned by Hsaio and Chen [50] and O’Connor et al [22] were included to accommodate the EMR utilization and health care context in Taiwan. In this study, we excluded self-efficacy and technology trust from the user characteristics reported by O’Connor et al [22] because self-efficacy was reported to be a nonsignificant factor affecting HCPs’ IT acceptance [62,63]. EMRs have been developed, used (not mandatorily), and incorporated into daily procedures in Taiwanese hospitals for several years; thus, technology trust was not considered in this model. In addition, personal innovativeness of IT and technostress were added as variables in the research model. Rai et al [64] indicated that personal innovativeness positively and significantly affected mHealth usage intention and assimilation. Technostress is an emergent problem derived from the pervasive use of technologies, which may significantly affect individual productivity and daily life [65]. We considered that EMR use in its infusion stage may be affected by technostress exerting an effect on EMR infusion and individual performance. However, the effects of personal innovativeness and technostress on EMR infusion and performance should be empirically validated. Thus, the research model (Figure 1) involved nine EMR infusion antecedents: accessibility, maturity, portability (technology), time criticality, interdependence, mobility (task), EMR infusion, and individual performance (the outcomes of EMR infusion). The measurement, operational definition, and number of items for the variables investigated in this study are summarized in Table 1.

The technology characteristics examined in this study included accessibility (availability), maturity, and portability; these factors determine the functionality or usability of an EMR. The task characteristics examined in this study included time criticality, interdependence, and mobility. The user characteristics examined in this study included personal innovativeness in IT, technostress, and habit. These characteristics represent individual traits and perceptions toward the use of IT.

**Table 1:** Measurement and operational definitions of variables.

Construct			Operational definition		References		Number of items
**Technology characteristics**							
	Accessibility	The ability of accessing EMR^a^ information when required		Gebauer et al [66], Liang et al [67]		3	
	Maturity	The existence of a level of EMR quality that is perceived as satisfactory and the perceived need for system improvement by the user		O’Connor et al [22], Liang et al [67]		5	
	Portability	The degree of ease associated with transporting the EMR		O’Connor et al [22], Gebauer et al [66]		3	
**Task characteristics**							
	Time criticality	The urgency when accessing information through the EMR		O’Connor et al [22], Liang et al [67]		3	
	Interdependence	The degree to which completing tasks using the EMR requires interaction with other people		Hsiao and Chen [45], Gebauer et al [66]		3	
	Mobility	The extent to which a task is being performed in different locations using the EMR		Gebauer et al [66], Liang et al [67]		3	
**User characteristics**							
	Personal innovativeness	Willingness to try out any new technology		Agarwal and Prasad [68], Thatcher and Perrewe [69]		4	
	Technostress	A problem of adaptation resulting from the health care professional’s inability to cope with EMR use in a healthy manner		Ragu-Nathan et al [70], Tu et al [71]		5	
	Habit	The extent to which an individual tends to use the EMR automatically		Limayem et al [72], Venkatesh et al [73]		3	
	EMR infusion	The extent of EMR infusion related to the exploratory, integrative, and future use of EMR		O’Connor et al [22], Tennant et al [23]		9	
	Individual performance	The extent to which EMR use (continuance) can improve the health care professional’s efficiency, effectiveness, and quality of medical activities		Junglas et al [74]		8	

^a^EMR: electronic medical record.

EMR infusion is the extent of the exploratory, integrative, and future use of the EMR. Individual performance is defined as the improvement in HCPs’ efficiency, effectiveness, and quality of medical activities through EMR continuance (use). O’Connor et al [22] and Tennant et al [23] reported that IT assimilation and infusion are two types of uses that are beyond routine use and refer to a deeper level of usage that enhances work tasks and performance. Technology, task, and user characteristics are considered key antecedents that affect IS infusion or continuance [22,23,50] and the extent of IS infusion can affect individual performance. However, not all technology, task, and user characteristics examined by Hsaio and Chen [50] were reported to be significant antecedents, implying that the factors affecting IS infusion may vary among different technology, task, and user groups. Thus, the antecedents of EMR infusion should be empirically examined and tested for long-term EMR system evaluation. We proposed four research hypotheses with nine subhypotheses, which are summarized in Textbox 1.

**Research hypotheses and subhypotheses.**

**H1: The technology characteristics of health care professionals (HCPs) significantly affect electronic medical record (EMR) infusion**

- H1a: The accessibility of an EMR significantly affects EMR infusion
- H1b: The maturity of an EMR significantly affects EMR infusion
- H1c: The portability of an EMR significantly affects EMR infusion

**H2: The task characteristics of HCPs significantly affect EMR infusion**

- H2a: Task time criticality significantly affects EMR infusion
- H2b: Task interdependence significantly affects EMR infusion
- H2c: Task mobility significantly affects EMR infusion

**H3: The user characteristics of HCPs significantly affect EMR infusion**

- H3a: Personal innovativeness in information technology significantly affects EMR infusion
- H3b: Technostress significantly affects EMR infusion
- H3c: Individual habit significantly affects EMR infusion

**H4: EMR infusion significantly affects individual performance**

### Instrument and Respondents

The respondents were HCPs (doctors and nurses) who worked at different departments in the case hospital, and we elaborated rigorous instrument design processes to minimize the potential risks of common method bias derived from a single-respondent questionnaire-based survey as reported by Podsakoff et al [75]. In the first stage, we established 50 preliminary measurement items by referencing the literature and validated the instrument to ensure its appropriateness for the purpose of this study. Individual performance was measured using 8 items adapted from the study of Junglas et al [74]. EMR infusion was assessed with 9 items adapted from the studies of O’Connor et al [22] and Tennant et al [23]. Technology characteristics, namely accessibility, maturity, and portability, were measured using 11 items adapted from studies conducted by Gebauer et al [66], Liang et al [67], and O’Connor et al [22]. Task characteristics, namely time criticality, interdependence, and mobility, were measured using 9 items adapted from Gebauer et al [66], Hsiao and Chen [45], Liang et al [67], and O’Connor et al [22]. User characteristics, namely personal innovativeness, technostress, and habit, were measured using 12 items adapted from Agarwal and Prasad [68], Limayem et al [72], Ragu-Nathan et al [70], Thatcher and Perrewe [69], Tu et al [71], and Venkatesh et al [73].

The second stage of the questionnaire design focused on the evaluation and selection of the measurement items. To evaluate the content validity of the questionnaire, the content validity index (CVI) was calculated, and a threshold value of >0.80 was recommended [76]. Two experts on EMRs and one health informatics professional were invited to examine the content validity of the instrument. One item in habit was excluded because its value was <0.80, and the average CVI of the questionnaire was 0.98. Ultimately, a final research questionnaire consisting of 49 items was developed, and each item of the questionnaire was scored on a 5-point Likert scale (1 for strongly disagree and 5 for strongly agree). The detailed description of the research instrument can be found in Multimedia Appendix 1. The questionnaire consisted of two major parts. The first part focused on the demographic data of respondents, namely age, sex, educational level, and experience using the EMR in the hospital. The second part included measurement items related to the antecedents of EMR, EMR infusion, and individual performance.

To ensure that the data collected were in line with the research objectives, only HCPs with >6 months experience using the EMR in the case hospital were included as participants in this study. A total of 120 doctors and 500 nurses from hospitals in southern Taiwan were recruited. The case hospital has been using EMRs since 2009 to improve their quality of care, continuity, safety, efficiency, and medical decision-making. In addition, the case hospital obtained the EMR accreditation issued by the Ministry of Health and Welfare of Taiwan. This certification indicates that the core EMR functionality can provide the services of cross-hospital health information exchange and EHRs with the support of the existing IT and IS infrastructure.

### Model Evaluation

#### Measurement Model

The collected data were further analyzed using WarpPLS 5.0 [77], with the partial-least squares (PLS) approach to perform extensive, scalable, and flexible casual modeling [78]. Chin [79] suggested using the PLS technique to analyze measurement and structural models. Several common model data fit and quality indices are recommended for WarpPLS because of their advantages compared with other variance-based structural equation modeling (SEM) methods, including the average path coefficient (APC), average *R^2^* (ARS), average adjusted *R^2^* (AARS), average block variance inflation factor (AVIF), average full collinearity variance inflation factor (AFVIF), Tenenhaus goodness of fit (GoF), and *R^2^* contribution ratio (RSCR) [77]. Kock [77] indicated that the addition of latent variables into a model can increase the ARS value but reduce the APC value; however, both ARS and APC can increase concurrently only when the addition of variance can improve the predictive and explanatory qualities of the overall model. The AARS is used to correct inappropriate increases in *R^2^* coefficients when predictors cannot adequately improve the explanatory value of each latent variable [77]. Both AVIF and AFVIF are often used to evaluate the collinearity of a model if new latent variables are added. Kock [80] mentioned that if all variance inflation factors resulting from a full collinearity test are equal to or lower than 3.3, the model can be considered free of common method bias for PLS-SEM. The GoF is an index used to evaluate the explanatory power of a model, and RSCR is used to examine the degree to which a model is free from negative *R^2^* effects [77].

#### Structural Model

WarpPLS 5.0 with the bootstrap resampling method was used to analyze the structural model, which was mainly evaluated using the path coefficient (*β*) and *R^2^* value. Path coefficients represent the strength and direction of the relationship between variables, and they are meaningful to research if they achieve a statistically significant level. *R^2^* values indicate the total variance of dependent variables explained by influencing variables, demonstrating the predictive power of the investigated model.

### Ethical Considerations

To address potential ethical concerns, our study protocol and informed consent forms were reviewed and approved by the institutional review board (IRB) of St. Martin De Porres Hospital in Taiwan before the distribution and collection of surveys. After receiving approval from the IRB of the target hospital (IRB-15B-021), we obtained voluntary and verbal consent from the study participants. The requirement to document consent was waived. The responses of HCPs were anonymous and unidentified.

## Results

### Demographic Data and Descriptive Statistics

The questionnaire was administered to collect data from HCPs who worked at the case hospital at the time of the survey and had at least 6 months of experience in using EMRs. A total of 316 questionnaires were distributed and 211 complete copies were returned, resulting in a valid response rate of 66.8%. Voluntary participation might explain the relatively high response rate. According to the collected demographic data (Table 2), most of the respondents were aged <40 years, were women, and had a bachelor’s or higher degree. Furthermore, over 70% of the respondents worked in the nursing department and the remaining respondents worked in the medical department. In addition, 93.4% (203/211) of the respondents had >1 year of experience in using EMRs, indicating the representativeness of the participants. Among the investigated demographic variables, experience in using the EMR and respondents’ department were found to be significantly related to EMR infusion and performance responses based on analysis of variance. This is reasonable because doctors are heavy EMR users, and users with abundant experience in using EMRs tend to have more positive attitudes toward exploratory, integrative, and future EMR use, and a better performance evaluation.

**Table 2:** Demographic characteristics of the respondents (N=211).

Characteristic		Respondents, n (%)
**Age (years)**		
	<30	73 (34.6)
	31-40	91 (43.1)
	41-50	31 (14.7)
	51-60	11 (5.2)
	> 60	5 (2.4)
**Gender**		
	Male	52 (24.7)
	Female	159 (75.3)
**Education level**		
	Junior college	29 (13.8)
	Bachelor	169 (80.1)
	Master (or higher)	13 (6.1)
**Experience in hospital (years)**		
	<1	8 (3.8)
	1-3	42 (19.9)
	3-6	56 (26.6)
	6-9	36 (17.0)
	>9	69 (32.7)
**Experience in using EMRs^a^** **(years)**		
	<1	14 (6.6)
	1-3	102 (48.4)
	3-5	60 (28.4)
	>5	35 (16.6)
**Department**		
	Nursing	150 (71.1)
	Medical	61 (28.9)

^a^EMR: electronic medical record.

Table 3 shows the descriptive statistics of responses to the original questionnaire, including 49 items of the 11 investigated constructs used in this study. Among the constructs, the mean scores were the highest for time criticality, followed by performance, interdependence, portability, habit, technostress, mobility, accessibility, EMR infusion, maturity, and personal innovativeness in IT. Time criticality, performance, and interdependence were the top three variables and had mean scores greater than 4.0, whereas personal innovativeness in IT had the lowest mean score of the investigated variables. This implied that participating HCPs have a more positive evaluation toward EMR use for supporting their task characteristics (time criticality and interdependence); however, they do not demonstrate excellent personal innovativeness in IT. We further evaluated the item appropriateness and consistency of the measured constructs among domain experts. In total, 42 items were used for further PLS-SEM analysis and 7 items were excluded.

**Table 3:** Descriptive statistics of constructs and their respective items.

Construct		Score, mean (SD)	Range
**ACC^a^**		3.85 (0.65)	1-5
	ACC1	3.94 (0.55)	2-5
	ACC2	3.83 (0.66)	2-5
	ACC3	3.77 (0.73)	1-5
**POR^b^**		3.96(.64)	2-5
	POR1^c^	4.10 (0.68)	2-5
	POR2	3.88 (0.64)	2-5
	POR3	3.91 (0.61)	2-5
**MAT^d^**		3.78 (0.67)	1-5
	MAT1	3.68 (0.71)	2-5
	MAT2	3.64 (0.76)	1-5
	MAT3	3.85 (0.60)	2-5
	MAT4^c^	3.88 (0.61)	2-5
	MAT5	3.85 (0.60)	2-5
**TC^e^**		4.05 (0.62)	1-5
	TC1^c^	3.89 (0.66)	1-5
	TC2	4.08 (0.60)	2-5
	TC3	4.18 (0.61)	2-5
**INT^f^**		4.02 (0.65)	2-5
	INT1	4.09 (0.65)	2-5
	INT2	4.02 (0.64)	2-5
	INT3	3.96 (0.67)	2-5
**MOB^g^**		3.89 (0.66)	1-5
	MOB1	3.91 (0.71)	1-5
	MOB2	3.93 (0.60)	2-5
	MOB3	3.83 (0.68)	2-5
**PI^h^**		3.60 (0.72)	1-5
	PI1	3.48 (0.70)	1-5
	PI1	3.43 (0.75)	1-5
	PI3^c^	3.72 (0.73)	2-5
	PI4^c^	3.76 (0.70)	2-5
**TS^i^**		3.90 (0.67)	2-5
	TS1	3.90 (0.71)	2-5
	TS2	3.90 (0.61)	2-5
	TS3	4.10 (0.64)	2-5
	TS4	4.15 (0.62)	2-5
	TS5^c^	3.47 (0.78)	2-5
**HAB^j^**		3.96 (0.57)	3-5
	HAB1	4.02 (0.56)	3-5
	HAB2	3.93 (0.57)	3-5
	HAB3	3.93 (0.59)	3-5
**INF^k^**		3.82 (0.64)	1-5
	INF1 (EU^l^1)	3.71 (0.69)	2-5
	INF2 (EU2	3.62 (0.71)	2-5
	INF3 (EU3)	3.64 (0.66)	2-5
	INF4 (IU^m^1)	3.97 (0.60)	2-5
	INF5 (IU2)	3.91 (0.60)	3-5
	INF6 (IU3)	3.96 (0.58)	3-5
	INF7 (FU^n^1)	3.97 (0.65)	2-5
	INF8 (FU2)	4.00 (0.61)	2-5
	INF9 (FU3)^c^	3.59 (0.67)	1-5
**PER^o^**		4.05 (0.58)	2-5
	PER1	4.20 (0.59)	3-5
	PER2	4.15 (0.55)	3-5
	PER3	4.02 (0.56)	3-5
	PER4	4.00 (0.57)	3-5
	PER5	4.00 (0.53)	3-5
	PER6	4.06 (0.56)	3-5
	PER7	4.03 (0.61)	3-5
	PER8^c^	3.94 (0.63)	2-5

^a^ACC: accessibility.

^b^POR: portability.

^c^Excluded from further analysis.

^d^MAT: maturity.

^e^TC: time criticality.

^f^INT: interdependence.

^g^MOB: mobility.

^h^PI: personal innovativeness in information technology.

^i^TS: technostress.

^j^HAB: habit.

^k^INF: electronic medical record infusion.

^l^EU: exploratory use.

^m^IU: integrative use.

^n^FU: future use.

^o^PER: performance.

### Measurement Model

As shown in Table 4, the results demonstrated that all of the model data fit and quality indices met the criteria suggested by Kock [77]. All of the APC, ARS, and AARS values exceeded the recommended values, thereby indicating a highly satisfactory fit. The AVIF and AFVIF values indicated the absence of the collinearly problem in the model, demonstrating that the model can be considered free of common method bias as suggested by Kock [80]. The GoF value also exceeded the suggested value, indicating a satisfactory fit, and the RSCR value indicated an excellent fit. These findings validated the data fit and quality indices of the EMR infusion model.

**Table 4:** Model fit and quality indices.

Fit indices	Value	Criteria	Result
Average path coefficient	0.193 (P<.001)	P<.05	Fit
Average *R*^2^	0.529 (P<.001)	P<.05	Fit
Average adjusted *R*^2^	0.521 (P<.001)	P<.05	Fit
Average block VIF^a^	1.954	Acceptable if ≤5, ideally ≤ 3.3	Fit
Average full collinearity VIF	2.324	Acceptable if ≤5, ideally ≤3.3	Fit
Tenenhaus GoF^b^	0.618	Small, ≥0.1; medium, ≥0.25; large, ≥0.36	Fit
*R*^2^ contribution ratio	1.000	Acceptable if ≥0.9, ideally=1	Fit

^a^VIF: variance inflation factor.

^b^GoF: goodness of fit.

We further evaluated the reliability and validity (convergent and discriminant validity) of the instrument as suggested by previous studies [79,81,82]. Hair et al [82] suggested evaluating the reliability and internal consistency of each construct as reflective indicators by performing principal component analysis. They recommended a cut-off value of >.70 for Cronbach α and composite reliability (CR). Furthermore, Fornell and Larcker [81] indicated that the value of average variance extracted (AVE) should exceed 0.5, and each square correlation should have adequate convergent and discriminant validity. As shown in Table 5, the Cronbach α (>.798), CR (>0.734), and AVE (>0.604) values of all constructs exceeded the recommended cut-off values, indicating satisfactory reliability and convergent validity. One criterion for adequate discriminant validity is that the square root of the AVE for each construct should exceed the correlation coefficient between the construct and other constructs in the research model [79]. All square roots of AVE values in this study (diagonals in Table 5) were higher than the correlation coefficients (off-diagonals in Table 5), indicating satisfactory discriminant validity. These findings demonstrated the adequate reliability, convergent validity, and discriminant validity of the model.

**Table 5:** Correlations among variables, and the reliability and validity of the research model.

Variables	Correlations^a^												AVE^b^ (>0.5)		CR^c^ (>0.7)		Cronbach α (>.7)
	ACC^d^	POR^e^	MAT^f^	TC^g^	INT^h^	MB^i^	TS^j^	PI^k^	HB^l^	INF^m^	PER^n^						
ACC	0.792	0.686	0.643	0.582	0.581	0.501	0.382	0.598	0.412	0.550	0.456	0.628		0.761		.830	
POR	0.686	0.830	0.715	0.538	0.414	0.374	0.439	0.481	0.619	0.663	0.576	0.689		0.753		.822	
MAT	0.643	0.715	0.787	0.546	0.480	0.321	0.370	0.375	0.449	0.538	0.460	0.619		0.801		.870	
TC	0.582	0.538	0.546	0.899	0.653	0.576	0.482	0.294	0.430	0.518	0.498	0.808		0.871		.855	
INT	0.581	0.414	0.480	0.653	0.856	0.658	0.429	0.336	0.309	0.517	0.447	0.732		0.857		.881	
MB	0.501	0.374	0.321	0.576	0.658	0.831	0.537	0.443	0.295	0.570	0.321	0.691		0.823		.868	
TS	0.382	0.439	0.370	0.482	0.429	0.537	0.805	0.353	0.531	0.635	0.453	0.812		0.875		.866	
PI	0.598	0.481	0.375	0.294	0.336	0.443	0.353	0.901	0.318	0.499	0.314	0.648		0.827		.798	
HB	0.412	0.619	0.449	0.430	0.309	0.295	0.531	0.318	0.865	0.675	0.704	0.749		0.899		.925	
INF	0.550	0.663	0.538	0.518	0.517	0.570	0.635	0.499	0.675	0.777	0.803	0.604		0.734		.801	
PER	0.456	0.576	0.460	0.498	0.447	0.321	0.453	0.314	0.704	0.662	0.803	0.645		0.892		.931	

^a^The values in the diagonal are square roots of the AVE and the off-diagonal elements are the correlation coefficients (*r*) among constructs.

^b^AVE: average variance extracted.

^c^CR: composite reliability.

^d^ACC: accessibility.

^e^POR: portability.

^f^MAT: maturity.

^g^TC: time criticality.

^h^INT: interdependence.

^i^MOB: mobility.

^j^PI: personal innovativeness in information technology.

^k^TS: technostress.

^l^HAB: habit.

^m^INF: electronic medical record infusion.

^n^PER: performance.

### Structural Model

**Figure 2:**
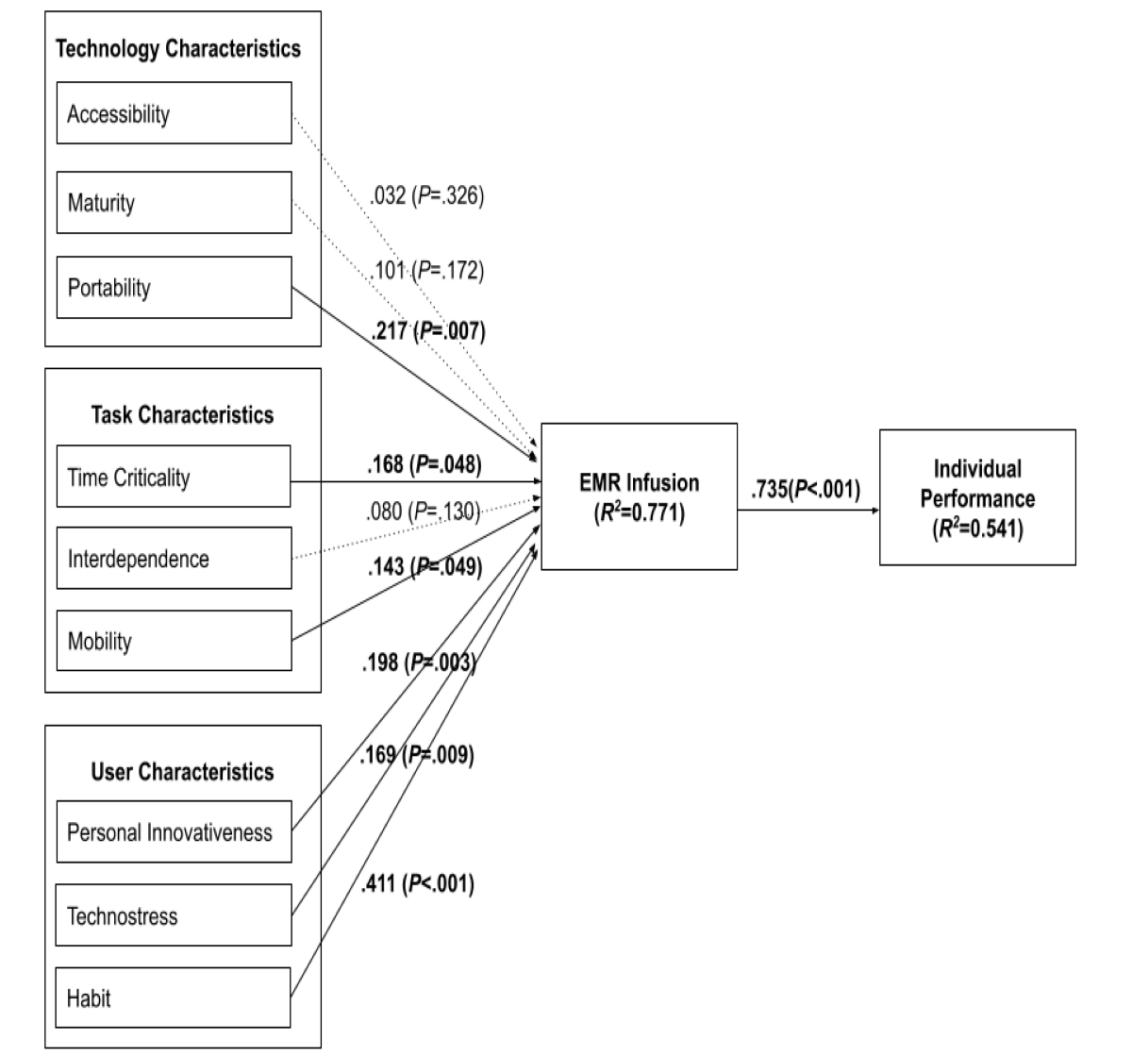
Results of model validity.

As shown in Figure 2, among the four major hypotheses (including nine subhypotheses) of this study, those related to technology characteristics (only H1c was positively supported) and task characteristics (only H2a and H2c were positively supported) were partially supported. However, all of the subhypotheses (H3a, H3b, and H3c) related to user characteristics were significantly supported. The results revealed that EMR infusion was mainly affected by portability among technology characteristics; time criticality and mobility among task characteristics; and personal innovation, technostress, and habit among user characteristics. Habit (H3a), portability (H1c), personal innovativeness (H3a), technostress (H3b), time criticality (H2a), and mobility (H2c) were identified as key factors affecting EMR infusion according to their relative effects on EMR infusion by ranking. In addition, individual performance was significantly affected by EMR infusion (H4). We further examined the direct effects, indirect effects, and total effects of the research variables, specifically for the mediation analysis of technology, task, and user characteristics (through infusion) on performance. As shown in Table 6, technostress, habit, personal innovativeness in IT, and mobility had significant mediating (indirect) effects through infusion on EMR performance.

**Table 6:** Direct, indirect, and total effects (β values) of research variables.

Variable			TS^a^		HAB^b^		INT^c^		TC^d^		MOB^e^		MAT^f^		POR^g^		ACC^h^		PI^i^		INF^j^
INF (direct effect)			.169 (P=.005)		.411 (P<.001)		.141		.031		.145		.059		.217		.047		.110		N/A^k^
**PER^l^**																					
	Direct effect	N/A		N/A		N/A		N/A		N/A		N/A		N/A		N/A		N/A		.735 (P<.001)	
	Indirect effect	.124 (P=.03)		.302 (P=.001)		.104 (P=.04)		–.023		.106 (P=.04)		.043		.159		–.034		.081		N/A	
	Total effect	.124 (P=.005)		.302 (P=.001)		.104		.023		.106		.043		.159		.034		.081		.735 (P<.001)	

^a^TS: time criticality.

^b^HAB: habit.

^c^INT: interdependence.

^d^TC: time criticality.

^e^MOB: mobility.

^f^MAT: maturity.

^g^POR: portability.

^h^ACC: accessibility.

^i^PI: personal innovativeness in information technology.

^j^INF: electronic medical record infusion.

^k^N/A: not applicable.

^l^PER: performance.

## Discussion

### Principal Findings

The results revealed that EMR infusion (*R^2^*=0.771) was mainly affected by user habits (*β*=.411), portability (*β*=.217), personal innovativeness (*β*=.198), technostress (*β*=.169), and time criticality (*β*=.168), whereas individual performance (*R^2^*=0.541) was affected by EMR infusion (*β*=.735). This finding indicated that user (habit, personal innovativeness, and technostress), technology (portability), and task (mobility and time criticality) characteristics have major influences on EMR infusion. Furthermore, the results indicated that EMR infusion positively affected individual performance. Consistent with the findings of previous IS infusion and TTF studies [10,21-23,50], EMR infusion was found to be affected by technology, task, and user (individual) characteristics. However, not all investigated technology, task, and user characteristics were found to be significant for EMR infusion. The results of this study revealed that among technology characteristics, only portability (*β*=.217) affected EMR infusion; however, the effects of accessibility and maturity on EMR infusion were not as expected. Portability is the degree of ease associated with transporting EMRs [22,66]. This finding is in accordance with the result reported by O’Connor et al [22] but is not consistent with that reported by Hsaio and Chen [50] for mHealth infusion. EMRs are computerized medical information systems that collect, store, and display patient information [2]; thus, they can be easily transported to HCPs to fulfill their needs. This finding may explain why portability was found to be a significant factor affecting EMR infusion.

O’Connor et al [22] reported that task characteristics, namely time criticality, interdependence, and mobility, were considered to be salient factors affecting mHealth infusion; however, Hsaio and Chen [50] found that mobility was the only significant factor affecting mHealth continuance. We found that among task characteristics, time criticality (*β*=.168) and mobility (*β*=.143) affected EMR infusion. The tasks of HCPs are complex and time-critical, and require mobility, specifically for providing services in inpatient and emergency departments. Real-time and accurate information obtained from an EMR is critical to increase efficiency and effectiveness in patient care duties [45]. These findings explain why time criticality and mobility were significant factors affecting EMR infusion. Consistent with the results of Hsaio and Chen [45,50], interdependence was found to be insignificant for EMR infusion.

This study found that user characteristics, namely personal innovativeness (*β*=.198), technostress (*β*=.217), and habit (*β*=.411), significantly affect EMR infusion. All three user characteristics exerted significantly stronger effects compared with those of technology and task characteristics on EMR infusion. This finding implied that user characteristics are the key antecedents of EMR infusion. The individual habit of EMR use showed a consistent result with the findings of O’Connor et al [22] and Hsaio and Chen [50]; however, personal innovativeness was not observed to be a significant factor in the context of mHealth [50]. Previous studies have reported that habit can affect future behavior if technology use becomes a habit as routine behavior [83,84], which is consistent with the findings of this study. The result of personal innovativeness in this study is also in accordance with that reported by Rai et al [64], who confirmed that personal innovativeness positively and significantly affected IS usage intention and assimilation. Consistent with the results reported by La Torre et al [65], we found that technostress significantly affected EMR infusion and individual productivity.

In this study, EMR infusion referred to the extent of EMR infusion related to the exploratory, integrative, and future use of EMRs, whereas individual performance was defined as the improvement in HCPs’ efficiency, effectiveness, and quality of medical activities through EMR continuance (use). Previous studies have indicated that IT assimilation and infusion are two types of use that are beyond routine use, and refer to a deeper level of usage that enhances work tasks and performance [22,23]. Consistent with findings of previous studies [22,23,50], this study indicated that EMR infusion significantly affected its outcomes (individual performance). We also found that technostress, habit, personal innovativeness in IT, and mobility have significant mediating (indirect) effects through EMR infusion on EMR performance. This implied that technostress, habit, and personal innovativeness in IT have both positive and significant direct effects on EMR infusion and indirect effects on EMR performance. Moreover, mobility was found to only have positive and significant indirect effects on EMR performance. Therefore, we should pay more attention to these significant factors of EMR infusion and performance.

We performed an additional analysis to determine HCPs’ performance based on EMR use. As shown in Table 3 (PER1-PER8), the top three items were as follows in descending order: PER1 (“EMR use accelerates information exchange with other members of the health care team”; score=4.20), PER2 (“EMR use reduces information retrieval time in clinical care practice”; score=4.15), and PER6 (“EMR use can make me more efficient at patient care”; score=4.06). Of all the items investigated, most respondents only provided a high and positive evaluation toward EMR performance, and only the mean value of the item “EMR use facilitates estimating and managing the costs of patient care” did not exceed 4 (mean 3.94, SD 0.63), indicating that this item had a slightly lower value than the other items. The results confirmed that EMR use can improve HCPs’ performance (mean 4.05, SD 0.58) related to efficiency, effectiveness, and quality of medical activities.

### Contributions to Medical Informatics Theory

Previous studies have suggested investigating the IS infusion chain, including the antecedents of IS infusion and the outcomes of IS infusion at the individual level with broader considerations, to examine the extent to which the full potential of ITs and ISs has been embedded in an organization’s or individual’s work system [22,23]. This study attempted to determine the reasons underlying the ceiling effect in EMR assimilation observed by Trudel et al [33]. From the theoretical perspective of TTF, the results of this study are in accordance with those reported by Goodhue and Thompson [10], who indicated that IT/IS can positively affect individual performance if the technology has been used continuously (utilization) and is a good fit for the supported task (TTF) in the EMR context. For the long-term evaluation of an IS, the fit among task, technology, and individual should be evaluated for the IS, and the IS should be continuously used for supporting the tasks. Thus, the IS infusion process can substantially affect individual performance. The appropriate, integrative, and exploratory use of EMRs in the infusion stage can significantly improve quality of care, continuity, safety, efficiency, and medical decision-making, and facilitate the exchange of cross-hospital health information and EHRs [1,2,4-9].

This is one of the few studies to specifically focus on EMR infusion by considering technology, task, and user aspects, and to examine EMR infusion effects on individual performance. The results of this study can be helpful for extending IS infusion research and identifying critical factors affecting EMR assimilation and infusion where EMRs have been deeply incorporated into the daily operating procedures of hospitals. In addition, EMR design and implementation should meet HCP task needs, particularly for time critically and mobility, and technology needs, specifically for EMR portability. Moreover, future studies can extend the results of this study by incorporating different behavioral theories and factors as the antecedents of IS infusion, and investigating their effects on IS infusion and individual performance. We found that accessibility (availability) and maturity among technology characteristics and interdependence among task characteristics were insignificant factors for EMR infusion. Therefore, additional studies should be performed to validate the factors investigated in the research model because their effects on IS infusion may vary depending on technology, task, and user groups. In addition, inspired by Kim et al [85] on what clinical information is valuable to doctors toward using a mobile EMR, we suggest that further studies pay attention to investigating the critical clinical information and functionalities of EMRs used by different HCPs in EMR infusion and to what extent they can effectively support their tasks with long-term technology use.

### Contributions to Medical Informatics Practice

The key factors identified in this study provide useful insights for the further improvement of EMR development in hospitals and the government, specifically for the infusion stage. In addition, the developed instrument can be used as an assessment tool for identifying the key considerations of EMR infusion, and for evaluating the extent of the EMR infusion and individual performance of hospitals that have implemented EMR systems. The results can help the government to understand the urgent needs of hospitals in implementing EMRs, provide sufficient resources and support for improving the incentives of EMR development, and develop adequate EMR policies for the widespread use of health information exchanges and EHRs. Future studies should focus attention on these characteristics, specifically user characteristics (personal innovativeness, technostress, and habit) and task characteristics (time critical and mobility), to further facilitate EMR infusion. Our findings indicate that the routine use of EMRs by HCPs in their daily workflow processes can reduce their technostress related to the use of EMRs and increase their perceived personal innovativeness, thus promoting EMR infusion.

### Limitations

This study has several limitations. First, we collected samples from a regional teaching hospital in Taiwan, restricting the generalization of the findings to other medical institutions. Second, the data were derived from questionnaires provided to participants with more than 6 months experience in using EMRs. Respondents answered questions based on their perceptions, experiences, and understanding. Thus, the data collected may not be adequately objective. However, due to the nature of this study (exploratory research), the quality of the collected data is acceptable. Furthermore, this study was based on a sample of voluntary participants. This type of recruitment is not considered to have negatively affected the results because this approach is commonly used in the field.

## Appendix

Multimedia Appendix 1 Questionnaire for EMR infusion and performance.

## Abbreviations

AARS: average adjusted R2
AFVIF: average full collinearity variance inflation factor
APC: average path coefficient
ARS: average R2
AVE: average variance extracted
AVIF: average block variance inflation factor
CR: composite reliability
CVI: content validity index
ECM: expectation confirmation model
EHR: electronic health record
EMR: electronic medical record
GoF: Tenenhaus Goodness of Fit
HCP: health care professional
HIS: health information system
HIT: health information technology
IRB: institutional review board
IS: information system
IT: information technology
mHealth: mobile health
PLS: partial least squares
RSCR: R2 contribution ratio
SEM: structural equation model
TAM: technology acceptance model
TTF: task-technology fit
UTAUT: unified theory of acceptance and use of technology
