# Supplementary material for: Health Professionals’ Perspectives on Electronic Medical Record Infusion and Individual Performance: Model Development and Questionnaire Survey Study
Source: JMIR Med Inform. 2021 Nov 30;9(11):e32180. doi: 10.2196/32180 (PMC8672292; doi:10.2196/32180)
Supplement: Multimedia Appendix 1 [file medinform_v9i11e32180_app1.docx]

## Appendix

### Questionnaire for EMR infusion and performance

1. ACC1: I can easily obtain support from EMR in clinical practices.
2. ACC2: I can use required EMR functions anytime under legal authorization and protection.
3. ACC3: EMR can be accessed anywhere in hospital to provide healthcare services.
4. **POR1*: EMR has the convenience of moving around in clinical practice compared with paper-based medical record.**
5. POR2: The availability of EMR is not a problem in clinical practice.
6. POR3: The use of EMR can satisfy the needs from different healthcare units in providing healthcare services.
7. MAT1: The reliability provided by EMR can satisfy my needs in clinical practices.
8. MAT2: The response time provided by EMR satisfy my needs in clinical practices.
9. MAT3: The data access function provided by EMR satisfy my needs in clinical practices.
10. **MAT4*: EMR has good mechanisms for information security control and management.**
11. MAT5: EMR provides all the functions required for performing my clinical work.
12. TC1: I often need to send various information to other medical personnel immediately when I am doing clinical practices.
13. TC2: I need to collect required healthcare information depending on different healthcare situations.
14. TC3: Providing immediate information reminders is important to me for performing my clinical work.
15. INT1: I often need to immediately control the patient in critical condition with other medical professionals.
16. INT2: I need to work with professionals to solve the clinical problems of the patient.
17. INT3: My clinical work is often cross-departmental.
18. MOB1: I often need to move in different locations when I do my clinical work.
19. MOB2: I need to constantly move in my workplace according to various situations when I am doing clinical work.
20. MOB3: The freedom to choose clinical work in a fixed working location is not high.
21. PI1: Others come to me for advice on new technology.
22. PI2: I am among the first in my circle of friends to acquire new technology.
23. **PI3*: I can figure out new high-tech products and services without any help.**
24. **PI4*: I can keep up with the latest technological development that I am interested in.**
25. TS1: I am forced to change my work habits to adapt the informatization in clinical practice.
26. TS2: I have to constantly update my knowledge and skills to information technologies.
27. TS3: I find it is an essential part of my job to use information technologies in my clinical work.
28. TS4: I find that information technologies have been widely used in the clinical work.
29. **TS5*: I often do not have enough time to study and upgrade my information technology skills in clinical practice.**
30. HAB1: The use of EMR has become a routine practice when providing healthcare services.
31. HAB2: The use EMR to perform clinical practice is my preferred way of performing tasks.
32. HAB3: Using the EMR has become automatic to me in my clinical work.
33. INF1: I will suggest my organization to implement additional EMR functions.
34. INF2: I often explore new functions provided by EMR.
35. INF3: I will use EMR as an innovative platform of innovative to deliver healthcare services.
36. INF4: I can provide my clinical services through the integrated information obtained from EMR.
37. INF5: I can arrange the priority of my clinical services according to the integrated information provided by EMR.
38. INF6: EMR can help me to coordinate required clinical services by the integrated information provided by EMR.
39. INF7: I will use all available functions provided by EMR in the future.
40. INF8: I will use most functions provided by EMR in the future.
41. **INF9*: I will use partial functions provided by EMR due to limitations of the system.**
42. PER1: The EMR use accelerates information exchange with other members of the health care team.
43. PER2: The EMR use reduces information retrieval time in clinical care practices.
44. PER3: The EMR use satisfies my needs in having sufficient clinical information when facing patients.
45. PER4: The EMR use improves medical quality.
46. PER5: The EMR use can be easier to perform clinical care activities.
47. PER6: The EMR use can make me more efficient patient care.
48. PER7: The EMR use allows me to follow the clinical guidelines more precise.
49. **PER8*: The EMR use facilitates estimating and managing the costs of patients care.**

***indicated items excluded for further analysis**

Note: ACC=Accessibility, POR=Portability, MAT=Maturity, TC= Time Criticality, INT=Interdependence, MOB=Mobility, INN= Personal Innovativeness in IT, TS=Technostress, HAB=Habit, INF=EMR Infusion, PER= Performance
